# Effect of Propofol Continuous-Rate Infusion on Intravenous Glucose Tolerance Test in Dogs

**DOI:** 10.3390/vetsci5020043

**Published:** 2018-04-20

**Authors:** Kenichi Maeda, Munehiro Iwasaki, Yuki Itou, Satomi Iwai, Shozo Okano

**Affiliations:** 1 Kitasato Laboratory of Small Animal Surgery 2, School of Veterinary Medicine, Kitasato University, Higashi 23-35-1, Towada, Aomori 034-8628, Japan; iwai@vmas.kitasato-u.ac.jp (S.I.); okano@vmas.kitasato-u.ac.jp (S.O.); 2 Aso Animal Hospital, 5-12-5 Hikino, Fukuyama, Hirosima 721-0942, Japan; m.i.5011.ahp@gmail.com; 3 Togasaki animal hospital, 3-528-1 Togasaki, Misato, Saitama 341-0044, Japan; yuuki748@yahoo.co.jp

**Keywords:** anesthesia, propofol, glucose metabolism, dog, free fatty acid

## Abstract

Hyperglycemia causes perioperative complications and many anesthetics impair glucose metabolism and cause hyperglycemia. We evaluated the effects of propofol on blood glucose metabolism and insulin secretion during an intravenous glucose tolerance test (IVGTT) in dogs. Blood glucose, insulin, triglyceride, cholesterol, and free fatty acid (FFA) levels were measured in dogs during IVGTT in a conscious state and under the effect of 2.0% isoflurane, low-concentration propofol (0.2 mg/kg/min), and high-concentration propofol (0.4 mg/kg/min) anesthesia. Plasma glucose levels significantly increased in all of the treatment groups when compared with those in the conscious group. The prolonged half-life period of plasma glucose suggested that isoflurane and propofol attenuated glucose metabolism in dogs. Plasma insulin levels were significantly lower in the isoflurane group when compared with those in the other groups, whereas blood FFA levels were increased in the propofol groups when compared with the other groups. These results suggest that propofol itself does not directly raise plasma glucose levels, but attenuates glucose metabolism by accumulating FFA.

## 1. Introduction

Several clinical studies have suggested that hyperglycemia increases the incidence of complications during the postoperative period in humans [1,2]. Even short-term hyperglycemia causes immunosuppression and is associated with significantly increased infections and patient mortality [2]. Some anesthetics are known to increase the incidence of hyperglycemia by suppressing glucose metabolism [3]. Propofol is one of these anesthetics and has been reported to suppress glucose metabolism in rats [4]. Propofol is a reliable and safe anesthetic agent. The favorable recovery profile associated with propofol offers advantages over traditional anesthetics in clinical situations where rapid recovery is important. Thus, propofol is now used in various situations, including general anesthesia induction, total intravenous anesthesia (TIVA), and sedation in critical care units. Furthermore, propofol is compatible with a large variety of pre-anesthetics that may increase its use as a safe and reliable intravenous anesthetic for the induction and maintenance of general anesthesia and sedation in small animal veterinary practice.

Propofol is mainly used to maintain general anesthesia with analgesics such as opioids and local anesthetics. TIVA with propofol may induce hyperglycemia and increase the risk of perioperative complications in glucose metabolism-suppressed patients including those with diabetes. Therefore, we hypothesized that the continuous rate injection of propofol may affect glucose metabolism in dogs, the same as in other species. However, to the best of our knowledge, there has been no study on the effects of propofol TIVA on the glucose metabolism in dogs. Hence, the aim of this study was to investigate the effect of propofol TIVA on glucose metabolism by monitoring the changes in plasma glucose and plasma insulin after 500 mg/kg glucose intravenous administration.

## 2. Materials and Methods

Seven healthy 4–5 year-old beagle dogs weighing 9.5–12.7 kg (four male, three female) were used in the present study. These dogs were bred solely for research purposes and were purchased from a licensed dealer. We performed blood tests for all of the dogs in this study, and certified the health condition of them, especially regarding plasma glucose, insulin, cholesterol, triglyceride, and free fatty acid before anesthesia induction. We set up at least seven days as the washout period for crossover study. All protocols in the present study were approved by the Institutional Animal Care and Use Committee of Kitasato University. All dogs were assigned to four experimental groups by using a 4 × 7 Latin square design: conscious dogs subjected to the intravenous glucose tolerance test (IVGTT) (Group A, *n* = 7); dogs under the effect of isoflurane anesthesia subjected to the IVGTT (Group S, *n* = 7); dogs under the effect of 0.2 mg/kg/min propofol anesthesia subjected to the IVGTT (Group 0.2 P, *n* = 7); and dogs under the effect of 0.4 mg/kg/min propofol anesthesia subjected to the IVGTT (Group 0.4 P, *n* = 7). Prior to the administration of the respective agents, the right cephalic vein was catheterized in all dogs using a 20-gauge catheter (B. Braun Vet Care, Tuttlingen, Germany) for the collection of venous blood samples, and the left cephalic vein was catheterized using a 22-gauge catheter for the administration of drugs and intravenous fluids (Solulact, Ringer Lactate Solution, Terumo, Tokyo, Japan). An additional 22-gauge catheter was placed in the right saphenous vein for continuous rate infusion (CRI) of propofol and fentanyl. In the anesthetized groups, dogs were fasted for 12 h before the induction of anesthesia. In Group A, dogs were also fasted for 12 h before the IVGTT was performed.

All dogs in Groups S, 0.2 P, and 0.4 P were pre-medicated with 0.05 mg/kg atropine sulfate hydrate (Mitsubishi Tanabe Pharma, Osaka, Japan) administered subcutaneously and 0.1 mg/kg midazolam (Dorumicum, Takeda Pharmaceutical, Tokyo, Japan) and 0.005 mg/kg fentanyl citrate (Fentanyl, Daiichi Sankyo, Osaka, Japan) administered intravenously before administering the general anesthesia. General anesthesia was induced with propofol (1% propofol inj. “Maruishi”, Maruishi Pharmaceutical, Osaka, Japan) at a dose of approximately 6 mg/kg, and tracheal intubation was performed. In Group S, as per the propofol groups, dogs were pre-medicated with 0.05 mg/kg atropine sulfate hydrate administered subcutaneously and 0.1 mg/kg midazolam and 0.005 mg/kg fentanyl citrate were administered intravenously before administering the general anesthesia. General anesthesia was induced with propofol at a dose of approximately 6 mg/kg. After 30-min maintenance of general anesthesia with 2.0% isoflurane (isoflurane for animal, Merck Animal Health, Madison, NJ, USA), the IVGTT was performed. In Groups 0.2 P and 0.4 P, after 30-min maintenance of general anesthesia with each dose of propofol continuous rate infusion (0.2 mg/kg/min or 0.4 mg/kg/min), the IVGTT was performed.

Baseline blood samples were collected before the glucose injection (0.5 g/mL glucose, Otsuka, Tokyo, Japan). A bolus of glucose solution (500 mg/kg in a 50% solution: 50% Otuska glucose injection, Otsuka Pharmaceutical, Tokyo, Japan) was given within 30 s into the left cephalic vein. After finishing the injection of glucose solution, we injected 1 mL saline as the flush solution. Additional blood samples were collected at 1, 5, 10, 20, 60, and 90 min from each dog, and the plasma was separated and stored at −20 °C until analysis. The glucose disappearance of the glucose load was obtained by plotting the blood glucose (at 5, 10, and 20 min) versus time on a semi logarithmic graph. A straight line was fitted to the data, from which the half-life of plasma glucose (T_1/2_) was determined. The glucose disappearance rate (KG) was obtained using the following formula: KG = 0.693/T_1/2_ × 100. To evaluate the insulin secretion against the glucose load, we calculated the insulinogenic index (IGI). IGI was computed as ΔInsulin 20/ΔGlucose 20 = (Insulin 20 min − Insulin 0 min)/(Glucose 20 min − Glucose 0 min) Furthermore, we obtained the blood glucose, insulin TG, and FFA curve and calculated the area under the curve (AUC) of glucose, insulin, TG, and FFA by time integrating over the period from 0 min to 90 min. General anesthesia was maintained for 2 h after intubation after which we stopped the isoflurane or propofol anesthesia and recovered. Under general anesthesia, normocapnia was maintained by controlled mechanical ventilation at an end-tidal carbon dioxide pressure ranging from 35 mmHg to 40 mmHg, and a heat blanket and heating pad were used to prevent hypothermia during the experiments. Blood plasma was analyzed for glucose, triglycerides (TG), and total cholesterol by using a blood biochemistry analyzer (Dimension RXLMax Integrated Chemistry System, Siemens Healthineers, Erlangen Germany). Insulin and free fatty acid (FFA) levels were analyzed by commercial laboratory services (Hoken Kagaku Kenkyujo, Yokohama, Japan) by using the chemiluminescence enzyme immunoassay and enzymatic method. The blood biochemistry analyzer was performed for accuracy control every three months using each standard reagent.

All data was normally distributed. The sample size calculation assumed the following: α value 0.05, power of 0.8. The calculated sample size consisted of seven dogs in each group, therefore, we decided that every experimental group should be composed of seven dogs. Statistical analysis was performed using the Tukey–Kramer test for intergroup comparison. The demographic variables and the baseline values were examined by one-way analysis of variance. Statistical significance was defined as *p* < 0.05. All data values were presented as means ± standard deviation. All the statistical analyses were performed with statistical software JMP (SAS Institute, Cary, NC, USA). 

## 3. Results

### 3.1. Plasma Glucose Level, Glucose Half-Life Period, and Disappearance Rate

Table 1 shows the time course of the mean plasma glucose, the glucose half-life time, and the disappearance rate for all groups, respectively. Plasma glucose levels increased after the administration of glucose in all groups. At 1 min after glucose administration, glucose levels were higher in Group S (24.0 ± 3.01 mmol/L), Group 0.2 P (23.5 ± 2.59 mmol/L), and Group 0.4 P (23.5 ± 1.53 mmol/L) when compared with the levels in Group A (19.7 ± 1.56 mmol/L). The blood glucose level started to decrease by the 5 min time point, and the glucose disappearance rate (KG) was significantly decreased in Group S (1.91 ± 0.23%/min), Group 0.2 P (2.07 ± 0.36%/min), and Group 0.4 P (2.20 ± 0.42%/min) when compared with the rate in Group A (3.41 ± 0.82%/min). Furthermore, the glucose half-life time (T_1/2_) was prolonged in Group S (36.7 ± 4.43 min), Group 0.2 P (34.3 ± 5.97 min), and Group 0.4 P (32.5 ± 5.91 min) when compared with the time in Group A (21.2 ± 4.31 min). There were no significant differences between the anesthetic groups pertaining to any of the measured parameters.

### 3.2. Plasma Insulin Levels

Table 2 shows the plasma insulin levels, which reached a maximum level at 1 min after glucose administration. In Group S, the maximum plasma insulin level was 25.0 ± 14.7 μIU/mL; at 5 min after glucose administration, plasma insulin level (14.7 ± 5.60 μIU/mL) was significantly lower than that in Group A (36.1± 11.7 μIU/mL) and Group 0.4 P (32.5 ± 14.1 μIU/mL). Furthermore, a significant difference was observed at 20 min (Group S versus Group 0.2 P) and 60 min (Group S versus Group A, Group S versus Group 0.4 P), whereas there were no significant differences in the area under the curve (AUC). The insulinogenic index (IGI) was 0.06 ± 0.01 in Group S, which was lower than those of Group A (0.21 ± 0.09) and Group 0.2 P (0.21 ± 0.14).

### 3.3. Plasma Cholesterol, TG, and FFA Levels

Table 3 shows the plasma cholesterol. There were no significant differences among all groups and time points. Table 4 shows the plasma TG levels. In the groups anesthetized with propofol, the plasma TG levels significantly increased after propofol CRI was initiated; 1 min after initiation, TG levels reached 0.85 ± 0.18 mmol/L in Group 0.2 P and 0.85 ± 0.26 mmol/L in Group 0.4 P. Increased TG levels in Groups 0.2 P and 0.4 P remained higher than those in Group A and Group S during the entire experimental period. Table 5 shows the FFA levels. The FFA levels, like the plasma TG levels, increased following propofol infusion. In particular, in Group 0.4 P, the plasma FFA levels reached 1.41 ± 0.54 mol/L after 5 min intubation; this significant difference was also observed at 20 min.

## 4. Discussion

Hyperglycemia in the perioperative period is due to stress hormones, such as epinephrine, cortisol, and inflammatory mediators. Even in the short-term, hyperglycemia causes immunosuppression and can be a risk factor for infectious complications and patient mortality [2]. Acute hyperglycemia during surgery worsens prognosis even in patients who returned normal glucose tolerance test results [5,6]. The effect of surgical stress on blood glucose is more prominent in patients with diabetes. Perioperative hyperglycemia is now known to be induced by not only surgical stress, but also anesthesia. Some studies have shown that inhalational anesthetics cause hyperglycemia in humans, rats, and dogs [3,4,7]. Furthermore, intravenous anesthetics, such as propofol, are also known to suppress glucose metabolism [8,9].

However, information about the effects of propofol on glucose metabolism is limited. There have been reports about the effects of general anesthetics on blood glucose metabolism, and the effects of propofol and inhalational anesthetic on glucose metabolism have been compared in a few human studies [9]. Inhalational anesthetics, such as isoflurane and sevoflurane have been reported to suppress blood glucose metabolism by attenuating insulin secretion from pancreatic beta cells [3,7,10]. Nevertheless, the effects of propofol on glucose metabolism have been insufficiently studied; in particular, there is no information on the modifying effects of propofol on glucose metabolism in dogs.

In this study, we evaluated glucose metabolism in dogs using the IVGTT. The IVGTT is an assessment of the first-phase insulin response to glucose for diagnosing glucose intolerance or overt diabetes in humans [11]. The IVGTT was undertaken to determine early insulin response after intravenous glucose administration and is suitable for directly evaluating insulin secretion from pancreatic beta cells and is widely applied for testing glucose metabolism in dogs [12].

The results of KG and T_1/2_ in this study indicated that isoflurane and propofol depressed the glucose disappearance from the blood stream and the plasma glucose level was kept higher than that of the conscious state. In agreement with previous reports, the depression of glucose disappearance by isoflurane was the result of low plasma insulin levels. Propofol, like inhalational anesthesia, is known to affect the blood glucose metabolisms by attenuating insulin secretion in humans [9] and rats [4].

In our results, isoflurane depressed IGI and we showed that isoflurane suppressed insulin secretion. On the other hand, propofol did not affect plasma insulin levels and IGI at any concentration, in other words, propofol did not affect insulin secretion; it is assumed that another mechanism underlies this depression of glucose disappearance.

As above-mentioned, propofol is used in several clinical situations; one of its reported side effects include propofol infusion syndrome (PRIS). Propofol is poorly soluble in water and is, thus, formulated in a white, oil-in-water emulsion [13]. Maruishi, the 1% propofol preparation we adopted, is an emulsion containing soybean oil, glycerol, and egg lecithin, which is the same as other propofol preparations. Soybean oil comprises several kinds of fat, mainly TG. A study has reported that these lipids could be a risk for PRIS, which is characterized by cardiac dysfunction, metabolic acidosis, renal failure, rhabdomyolysis, and hyperlipidemia [14]. High-doses or long-term propofol infusion is known to induce metabolic disorder in patients. Administered TG is metabolized to FFA, which is known to cause insulin resistance by suppressing GLUT-4 expression on skeletal muscle cell surfaces. Plasma FFA taken into skeletal muscle cells by CD 36 activates I kappa B kinase (IKK) via the protein kinase C. Furthermore, it activated IKK phosphorylate insulin receptor substrate 1 (IRS-1), suppressing the GLUT-4 transport from the cytoplasm to the cell surface [15,16,17]. Although we did not examine these signal transductions in the present study, we found that propofol CRI increased the blood TG levels. Additionally, the accumulation of FFA found in 0.4 P but not in 0.2 P showed its dose-dependent manner. This accumulation of FFA may be the result of the insufficient metabolic ability at a rate of 0.4 mg/kg/min. However, the suppression effect on glucose metabolism could be reduced by selecting an appropriate propofol preparation. For example, 2% propofol, which is currently used in human medicine, could prevent the accumulation of TG and FFA. Using a 2% propofol preparation could reduce the risk of hyperglycemia in dogs.

There are some limitations to this study. First, we studied healthy dogs without surgical stress; therefore, we could not determine the effect of propofol TIVA on the glucose metabolism of patients with diabetes during surgery. A further clinical study is needed to elucidate the effect of propofol TIVA in diabetic dogs. Second, we injected fentanyl with propofol to replicate a clinical situation; there may have been some interference by fentanyl. To the best of our knowledge, there have been no reports on opioids affecting glucose metabolism, but other drugs used in the perioperative period including benzodiazepines [18], alpha-adrenergic receptor agonists [19], and steroids [20] attenuate glucose metabolism. The lactate linger solution we injected in this study is usually used in the perioperative period to maintain blood pressure. Lactate is known to depress glucose metabolism in liver, so it is true that lactate may affect glucose metabolism. However, the effect of lactate on glucose is restrictive and we can exclude that effect in healthy dogs. Therefore, we should accumulate more information about the effects of other drugs and transfusions with propofol on glucose metabolism in dogs. Third, we concluded that FFA attenuated glucose metabolism, but we do not know how propofol itself may affect glucose metabolism. The effect of propofol on glucose metabolism in dogs should be evaluated in future studies. Furthermore, to replicate a clinical situation, we injected propofol for general anesthesia induction in Group S. There is a possibility that affecting this injection may depress glucose metabolism. However, our result showed that there were no changes observed in FFA and TG in Group S and we can exclude the effect of a single injection of propofol. Finally, we could not fully discuss the insulin secretions as we only monitored the change of plasma insulin levels and IGI, and did not measure insulin secretion and insulin clearance directly. C-peptide is generally found in amounts equal to insulin as insulin and C-peptide are linked when first made by the pancreas. The level of C-peptide in the blood can show how much insulin is being made by the pancreas. Therefore, the measurement of C-peptides would provide in-depth data in future studies.

## 5. Conclusions

In this study, our data showed that propofol TIVA depressed glucose disappearance from the blood stream without affecting plasma insulin secretion in dogs. Furthermore, we suggest that this depression was induced by accumulating FFA derived from injected lipids. From these results, we should consider this depression during the perioperative period. In particular, patients with diabetes should be strictly monitored for blood glucose, and particular attention should be paid when using propofol for patients with a known glucose metabolism disorder.

## Figures and Tables

**Table 1:** Changes in glucose concentrations after the glucose challenge, the area under the curve, the glucose disappearance rate (KG), and the half-life of plasma glucose (T_1/2_). Pre (before anesthesia induction): each time point indicates the elapsed time after the glucose challenge; 0 min means just before the glucose injection. Data are expressed as mean ± SD.

	Group A	Group S	Group 0.2 P	Group 0.4 P
	Plasma Glucose Levels (mmol/L)			
Pre	5.42 ± 0.52	5.54 ± 0.26	5.31 ± 0.31	5.49 ± 0.26
0 min	5.46 ± 0.25	5.31 ± 0.48	5.49 ± 0.45	5.53 ± 0.35
1 min	19.7 ± 1.56	24.0 ± 3.01 *	23.5 ± 2.59 ^†^	23.5 ± 1.53 ^‡^
5 min	16.7 ± 1.44	18.7 ± 2.18	17.9 ± 1.77	18.1 ± 1.37
20 min	10.0 ± 1.33	13.9 ± 1.75 *	12.8 ± 1.69 ^†^	13.2 ± 1.56 ^‡^
60 min	5.33 ± 0.34	8.72 ± 1.77 *	7.23 ± 1.80	7.45 ± 1.97
90 min	4.48 ± 1.83	6.66 ± 1.30	5.87 ± 1.30	6.48 ± 1.27
	AUC (×10^3^)			
	12.1 ± 1.47	18.0 ± 3.07 *	16.0 ± 2.88 ^†^	16.5 ± 2.56 ^‡^
	KG (%/min)			
	3.41 ± 0.82	1.91 ± 0.23 *	2.07 ± 0.36 ^†^	2.20 ± 0.42 ^‡^
	T_1/2_ (min)			
	21.2 ± 4.31	36.7 ± 4.43 *	34.3 ± 5.97 ^†^	32.5 ± 5.91 ^‡^

* *p* < 0.05 Group S versus Group A; ^†^ *p* < 0.05 Group 0.2 P versus Group A; ^‡^ *p* < 0.05 Group 0.4 P versus Group A.

**Table 2:** Changes in insulin concentrations after the glucose challenge, the area under the curve (AUC) and insulinogenic index (IGI). Pre (before anesthesia induction): each time point indicates the elapsed time after the glucose challenge; 0 min means just before the glucose injection. Data are expressed as mean ± SD.

	Group A	Group S	Group 0.2 P	Group 0.4 P
	Plasma Insulin Levels (µIU/mL)			
Pre	4.76 ± 1.15	4.22 ± 1.70	4.05 ± 2.98	4.69 ± 2.11
0 min	4.57 ± 2.00	0.65 ± 0.46	2.33 ± 1.53	2.29 ± 1.23
1 min	55.0 ± 23.9	25.0 ± 14.7	51.4 ± 19.8	53.8 ± 34.3
5 min	36.1 ± 11.7	14.7 ± 5.60 *^,††^	30.5 ± 10.4	32.5 ± 14.1
20 min	21.8 ± 7.31	12.7 ± 7.42 ^§^	28.4 ± 15.0	26.8 ± 10.3
60 min	2.74 ± 1.24	10.6 ± 5.38 *^,††^	9.04 ± 5.94	9.63 ± 4.41
90 min	3.34 ± 1.59	7.06 ± 5.46	6.01 ± 5.27	7.45 ± 4.24
	AUC (×10^3^)			
	1.26 ± 0.24	1.13 ± 0.46	1.69 ± 0.57	1.74 ± 0.36
	Insulinogenic Index			
	0.21 ± 0.09	0.06 ± 0.01 *^,§^	0.21 ± 0.14	0.19 ± 0.09

* *p* < 0.05 Group S versus Group A; ^††^ *p* < 0.05 Group S versus Group 0.4 P; ^§^ *p* < 0.05 Group S versus Group 0.2 P.

**Table 3:** Changes in cholesterol concentrations after the glucose challenge. Pre (before anesthesia induction): each time point indicates the elapsed time after the glucose challenge; 0 min means just before the glucose injection. Data are expressed as mean ± SD.

	Group A	Group S	Group 0.2 P	Group 0.4 P
	Plasma Cholesterol Levels (mmol/L)			
Pre	3.78 ± 1.00	3.72 ± 0.84	3.27 ± 1.28	3.72 ± 0.87
0 min	3.54 ± 0.95	3.26 ± 0.69	3.07 ± 1.20	3.42 ± 0.80
1 min	3.26 ± 0.85	2.94 ± 0.66	2.78 ± 1.12	3.11 ± 0.79
5 min	3.28 ± 0.87	2.97 ± 0.69	2.85 ± 1.15	3.18 ± 0.80
20 min	3.44 ± 0.93	3.02 ± 0.70	2.96 ± 1.19	3.33 ± 0.84
60 min	3.48 ± 0.88	3.02 ± 0.69	2.95 ± 1.19	3.35 ± 0.87
90 min	3.10 ± 1.58	2.92 ± 0.68	2.90 ± 1.15	3.51 ± 0.76

**Table 4:** Changes in triglyceride concentrations after the glucose challenge and the area under the curve (AUC). Pre (before anesthesia induction): each time point indicates the elapsed time after the glucose challenge; 0 min means just before the glucose injection. Data are expressed as mean ± SD.

	Group A	Group S	Group 0.2 P	Group 0.4 P
	Plasma Triglyceride Levels (mmol/L)			
Pre	0.40 ± 0.30	0.16 ± 0.06	0.23 ± 0.04	0.25 ± 0.17
0 min	0.39 ± 0.30	0.21 ± 0.07	0.86 ± 0.20 ^‡^^,§^	1.07 ± 0.29 ^†^^,††^
1 min	0.35 ± 0.31	0.19 ± 0.06	0.85 ± 0.18 ^‡^^,§^	0.85 ± 0.26 ^†^^,††^
5 min	0.38 ± 0.32	0.18 ± 0.06 *	0.77 ± 0.18 ^‡^^,§^	0.80 ± 0.25 ^†^^,††^
20 min	0.31 ± 0.30	0.19 ± 0.05	0.71 ± 0.22 ^‡^^,§^	0.78 ± 0.27 ^†^^,††^
60 min	0.23 ± 0.20	0.19 ± 0.05	0.61 ± 0.16 ^‡^^,§^	0.92 ± 0.34 ^†^^,††,¶^
90 min	0.26 ± 0.26	0.17 ± 0.06	0.53 ± 0.14 ^‡^^,§^	0.88 ± 0.20 ^†^^,††,¶^
	AUC			
	29.5 ± 18.9	19.5 ± 5.90	66.7 ± 11.9 ^‡^^,§^	89.9 ± 25.3 ^†,††^

* *p* < 0.05 Group S versus Group A; ^†^ *p* < 0.05 Group 0.4 P versus Group A; ^‡^ *p* < 0.05 Group 0.2 P versus Group A; ^††^ *p* < 0.05 Group S versus Group 0.4 P; ^§^ *p* < 0.05 Group S versus Group 0.2 P; ^¶^ *p* < 0.05 Group 0.2 P versus Group 0.4 P.

**Table 5:** Changes in free fatty acid concentrations after the glucose challenge and the area under the curve (AUC). Pre (before anesthesia induction): each time point indicates the elapsed time after the glucose challenge; 0 min means just before the glucose injection. Data are expressed as mean ± SD.

Time Point	Group A	Group S	Group 0.2 P	Group 0.4 P
	Plasma Free Fatty Acid Levels (mEq/L)			
Pre	0.70 ± 0.23	0.61 ± 0.18	0.71 ± 0.19	0.69 ± 0.13
0 min	0.72 ± 0.19	0.24 ± 0.13	0.58 ± 0.16	0.88 ± 0.18
1 min	0.70 ± 0.10	0.24 ± 0.10	0.60 ± 0.17	1.26 ± 0.51 ^†^
5 min	0.54 ± 0.11	0.25 ± 0.10	0.75 ± 0.20	1.41 ± 0.54 ^†^
20 min	0.17 ± 0.06	0.13 ± 0.08	0.34 ± 0.13	0.96 ± 0.50 ^†^
60 min	0.43 ± 0.14	0.05 ± 0.03	0.21 ± 0.10	0.52 ± 0.17
90 min	0.57 ± 0.27	0.06 ± 0.08	0.28 ± 0.18	0.65 ± 0.14
	AUC			
	43.9 ± 11.6 *	10.0 ± 5.97	34.4 ± 11.4 ^§^	81.3 ± 24.0 ^†^^,††,¶^

* *p* < 0.05 Group S versus Group A; ^†^ *p* < 0.05 Group 0.4 P versus Group A; ^††^ *p* < 0.05 Group S versus Group 0.4 P; ^§^ *p* < 0.05 Group S versus Group 0.2 P; ^¶^ *p* < 0.05 Group 0.2 P versus Group 0.4 P.

## References

[1] Ljungqvist O., Jonathan E. (2012). Rhoads lecture insulin resistance and enhanced recovery after surgery. J. Parenter. Enter. Nutr..

[2] Turina M., Miller F.N., Tucker C.F., Polk H.C. (2006). Short-term hyperglycemia in surgical patients and a study of related cellular mechanisms. Ann. Surg..

[3] Diltoer M., Camu F. (1988). Glucose homeostasis and insulin secretion during isoflurane anesthesia in humans. Anesthesiology.

[4] Sato K., Kitamura T., Kawamura G., Mori Y., Sato R., Araki Y., Yamada Y. (2013). Glucose use in fasted rats under sevoflurane anesthesia and propofol anesthesia. Anesth. Analg..

[5] Bochicchio G.V., Sung J., Joshi M., Bochicchio K., Johnson S.B., Meyer W., Scalea T.M. (2005). Persistent hyperglycemia is predictive of outcome in critically ill trauma patients. J. Trauma Acute Care Surg..

[6] Puskas F., Grocott H.P., White W.D., Mathew J.P., Newman M.F., Bar-Yosef S. (2007). Intraoperative hyperglycemia and cognitive decline after CABG. Ann. Thorac. Surg..

[7] Vore S.J., Aycock E.D., Veldhuis J.D., Butler P.C. (2001). Anesthesia rapidly suppresses insulin pulse mass but enhances the orderliness of insulin secretory process. Am. J. Physiol. Endocrinol. MeTab..

[8] Kitamura T., Ogawa M., Kawamura G., Sato K., Yamada Y. (2009). The effects of sevoflurane and propofol on glucose metabolism under aerobic conditions in fed rats. Anesth. Analg..

[9] Kitamura T., Kawamura G., Ogawa M., Yamada Y. (2009). Comparison of the changes in blood glucose levels during anesthetic management using sevoflurane and propofol. Masui. Jpn. J. Anesthesiol..

[10] Tanaka T., Nabatame H., Tanifuji Y. (2005). Insulin secretion and glucose utilization are impaired under general anesthesia with sevoflurane as well as isoflurane in a concentration-independent manner. J. Anesth..

[11] Bergman R.N. (1989). Toward physiological understanding of glucose tolerance: Minimal-model approach. Diabetes.

[12] Brunetto M.A., Sá F.C., Nogueira S.P., Gomes Mde O., Pinarel A.G., Jeremias J.T., de Paula F.J., Carciofi A.C. (2011). The intravenous glucose tolerance and postprandial glucose tests may present different responses in the evaluation of obese dogs. Br. J. Nutr..

[13] Barbosa F.T. (2007). Propofol infusion syndrome. Rev. Bras. Anestesiol..

[14] Bray R.J. (1998). Propofol infusion syndrome in children. Paediatr. Anaesth..

[15] Armoni M., Harel C., Bar-Yoseph F., Milo S., Karnieli E. (2005). Free fatty acids repress the GLUT4 gene expression in cardiac muscle via novel response elements. J. Biol. Chem..

[16] Van Epps-Fung M., Williford J., Wells A., Hardy R.W. (1997). Fatty acid-induced insulin resistance in adipocytes. Endocrinology.

[17] Yang S., Zhang W., Zhen Q., Gao R., Du T., Xiao X., Wang Z., Ge Q., Hu J., Ye P. (2015). Impaired adipogenesis in adipose tissue associated with hepatic lipid deposition induced by chronic inflammation in mice with chew diet. Life Sci..

[18] Yamada J., Sugimoto Y., Noma T. (2000). Involvement of adrenaline in diazepam-induced hyperglycemia in mice. Life Sci..

[19] Fagerholm V., Haaparanta M., Scheinin M. (2011). α2-adrenoceptor regulation of blood glucose homeostasis. Basic Clin. Pharmacol. Toxicol..

[20] Pandit M., Burke J., Gustafson A., Minocha A., Peiris A. (1993). Drug-induced disorders of glucose tolerance. Ann. Int. Med..

